# Rat bone marrow mesenchymal stem cells induced by rrPDGF-BB promotes bone regeneration during distraction osteogenesis

**DOI:** 10.3389/fbioe.2023.1110703

**Published:** 2023-03-07

**Authors:** Shuo Wu, Lijie Zhang, Ruidan Zhang, Kang Yang, Qin Wei, Qiyu Jia, Jian Guo, Chuang Ma

**Affiliations:** ^1^ Department of Microrepair and Reconstruction, the First Affiliated Hospital of Xinjiang Medical University, Urumqi, China; ^2^ Department of Neurology, the Second Affiliated Hospital of Xinjiang Medical University, Urumqi, China; ^3^ Guangdong New Omega Medical Centre, Guangzhou, China; ^4^ Hand and foot microsurgery of the third people’s Hospital of Xinjiang Uygur Autonomous Region, Urumqi, China; ^5^ Animal Experiment Center of Xinjiang Medical University, Urumqi, China

**Keywords:** BMSCs, distraction osteogenesis (DO), PDGF-BB, rat, osteogenesis, femur

## Abstract

**Background:** In the clinical treatment of large bone defects, distraction osteogenesis can be used. However, some patients may suffer from poor bone regeneration, or even delayed healing or non-union. Problems with the aggregation and proliferation of primary osteoblasts, or problems with the differentiation of primary osteoblasts will lead to poor bone regeneration. Therefore, supplementing exogenous primary osteoblasts and growth factors when using distraction osteogenesis may be a treatment plan with great potential.

**Methods:** Bone marrow mesenchymal stem cells (BMSCs) were extracted from rats and cultured. Subsequently, Recombinant Rat Platelet-derived Growth Factor BB (rrPDGF-BB) was used to induce bone marrow mesenchymal stem cells. At the same time, male adult rats were selected to make the right femoral distraction osteogenesis model. During the mineralization period, phosphate buffer salt solution (control group), non-induction bone marrow mesenchymal stem cells (group 1) and recombinant rat platelet-derived growth factor BB intervened bone marrow mesenchymal stem cells (group 2) were injected into the distraction areas of each group. Then, the experimental results were evaluated with imaging and histology. Statistical analysis of the data showed that the difference was statistically significant if *p* < 0.05.

**Results:** After intervention with recombinant rat platelet-derived growth factor BB on bone marrow mesenchymal stem cells, the cell morphology changed into a thin strip. After the cells were injected in the mineralization period, the samples showed that the callus in group 2 had greater hardness and the color close to the normal bone tissue; X-ray examination showed that there were more new callus in the distraction space of group 2; Micro-CT examination showed that there were more new bone tissues in group 2; Micro-CT data at week eight showed that the tissue volume, bone volume, percent bone volume, bone trabecular thickness, bone trabecular number and bone mineral density in group 2 were the largest, and the bone trabecular separation in group 2 was the smallest. There was a statistical difference between the groups (*p* < 0.05); HE staining confirmed that group 2 formed more blood vessels and chondrocytes earlier than the control group. At 8 weeks, the bone marrow cavity of group 2 was obvious, and some of them had been fused.

**Conclusion:** The study confirmed that injecting bone marrow mesenchymal stem cellsBB into the distraction space of rats can promote the formation of new bone in the distraction area and promote the healing of distraction osteogenesis.

## 1 Introduction

In the clinical practice, large bone defects can be treated with Ilizarov technology (Qin, 2021; Zheng et al., 2021). However, some patients had bone regeneration deficiency, which eventually led to delayed healing or even difficult healing (Borzunov and Shastov, 2019; Mi et al., 2021; Migliorini et al., 2021). Some scholars believe that the principle of poor bone regeneration is that there is a problem in the aggregation and proliferation of original osteoblasts, or there is a problem in the differentiation of original osteoblasts (Feng et al., 2016). Clinical studies have found that patients with primary osteoblast deficiency, severe trauma and severe tissue defects after radiotherapy will suffer from bone regeneration deficiency (Hamushan et al., 2020; Shen et al., 2021; Ye et al., 2021). Therefore, when using distraction osteogenesis to treat large bone defects, it may be a reasonable treatment plan to supplement exogenous original osteoblasts.

A large number of scholars have studied the principle of distraction osteogenesis. Some scholars reported that during distraction osteogenesis, BMSCs were recruited from the incubation period, and differentiated into osteoblasts or chondrocytes in the extended and mineralized periods (Ai-Aql et al., 2008; Dhaliwal et al., 2016), and a large number of callus was formed (Wang et al., 2013; Yuan-Zhe and Lee, 2018). Yang found that injecting mesenchymal stem cells into the distraction space can promote the formation of new bone tissue and shorten the mineralization time. Therefore, transplantation of BMSCs was a valuable regenerative therapy (Yang et al., 2017). However, the survival rate of BMSCs after transplantation was low, the differentiation was poor, and the regeneration ability was reduced (Ortinau et al., 2019). Some scholars proposed to enhance the activity of BMSCs through endogenous or exogenous methods to promote the proliferation of BMSCs, which may further promote bone repair.

Platelet-derived growth factor-BB (PDGF-BB) is considered to be a key regulator of tissue repair and regeneration. Studies have shown that PDGF-BB can promote cell mitosis (Gruber et al., 2003; Lee et al., 2013), chemotactic BMSCs and fibroblasts to reach the callus site (Heldin and Westermark, 1999; Ozaki et al., 2007), and promote tissue angiogenesis (Homsi and Daud, 2007). Some scholars reported that exogenous PDGF-BB was injected into the distraction space of rats, and the results showed that the volume of regenerated bone in the experimental group was significantly larger than that in the control group (Moore et al., 2009). Therefore, it was confirmed that PDGF-BB injection could promote the formation of new bone in the distraction space, accelerate the mineralization of bone tissue, and shorten the healing time. However, growth factor injection therapy has many disadvantages, such as the high price of growth factor, short and unstable half-life, easy infection caused by repeated injections, and difficult to grasp the injection dose and frequency. Therefore, it is particularly important to select an appropriate release control system. Our research group plans to inject the BMSCs induced by rrPDGF-BB into distraction space of rats to verify its osteogenic effect.

The main method of this study was to culture rat BMSCs *in vitro*, and use rrPDGF-BB to induce BMSCs. At the same time, the rat femur distraction osteogenesis model was made, and then the induced BMSCs were injected into the distraction space, then the experimental results were evaluated by imaging and histology. The results confirmed that the injection of BMSCs induced by rrPDGF-BB into the distraction space can promote the formation of new bone in the distraction area of rats. We expect that this study will promote the development of distraction osteogenesis technology.

## 2 Materials and methods

### 2.1 Experimental animals

120 male Sprague-Dawley (SD) rats, 48 of which were used to extract BMSCs (weight 120–150 g), 72 of which were used to build femoral distraction osteogenesis models (weight 350–400 g). The rats were purchased from Animal Experiment Center of Xinjiang Medical University [Certificate No: SYXK (Xin) 2018–0003]. The rats were raised in a standard and suitable environment for 7 days before surgery. The animal study was reviewed and approved by Ethics Committee of the First Affiliated Hospital of Xinjiang Medical University (NO. IACUC20190818-06). Written informed consent was obtained from the owners for the participation of their animals in this study.

### 2.2 Main instruments

**FIGURE 1:**
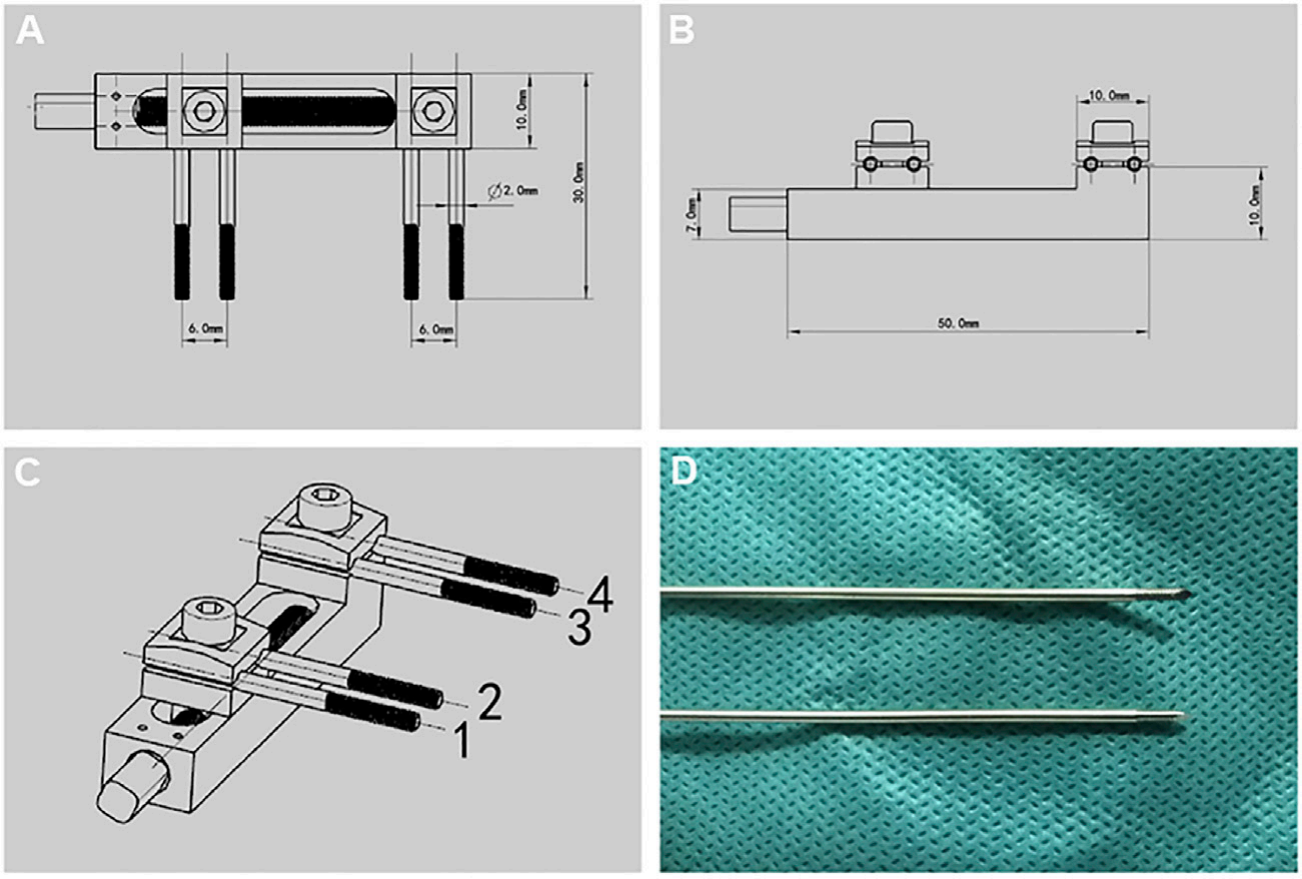
Design drawing of external fixation device and Kirschner wire. **(A-C)** Design drawing of external fixation device. **(D)** Threaded Kirschner wire matched with external fixation device.

Distraction osteogenesis external fixation device (Figure 1), designed by the key laboratory of the Institute of Engineering and Technology of Xinjiang University, mainly made of titanium alloy, 50.0 mm long, 10.0 mm wide, and 10.0 mm high; The end contains a rotating nut, which is used with an internal square wrench. The internal square wrench can be rotated 360° clockwise to increase the distraction space by 0.5 mm. External fixation device with four pieces of 1.0 × 200.0 mm self tapping threaded Kirschner wire is made of titanium alloy, with thread at the end and thread length of 12.0 mm. For the convenience of the following description, we mark the corresponding nail path of the external fixation device, which is marked by number 1–4 (Figure 1C).

### 2.3 Rat BMSCs culture

SD rats weighing 120–150 g were selected and anesthetized with 2% pentobarbital sodium (3 mg/100 g, Huaye Huanyu Chemical, Beijing, China). After the anesthesia took effect, the rats were killed by cervical dislocation; After soaking in 75% ethanol (Caoshanhu, Jiangxi, China) for 10 min, transfer it to the super clean table (Sujing, Shanghai, China, SM-CJ-1D), cut the skin and subcutaneous tissue of rats, take out bilateral tibia, fibula and femur, and soak them in sterile phosphate buffer salt solution (PBS, Gibco, United States) containing 1% penicillin (Gibco, United States), Use sterile surgical scissors (Wilson, Shanghai, China) to cut off both ends of the bone, use a 5.0 mL syringe to absorb low sugar complete culture medium to repeatedly wash the bone marrow cavity, blow the bone marrow solution evenly repeatedly and then centrifugate it (2000 r/min, 10 min), wash it twice with PBS, discard the upper liquid, blow the lower bone marrow solution evenly and then inoculate it in the culture bottle (Corning, United States), The low sugar complete medium (Gibco, United States) containing 12% fetal bovine serum (Gibco, United States) was used for culture.

### 2.4 Flow cytometry identification of rat BMSCs

The third generation BMSCs were cultured, and when they filled 90% of the bottom of the culture bottle, they were fully digested with 0.25% trypsin (Gibco, United States). Divide the cell fluid into four tubes, ensuring that each tube has at least 2 × 10^5^ cells were washed twice with PBS, then CD29-PE antibody (BD Biosciences, United States), CD34-PE antibody (Abcam, Cambridge, United Kingdom) and CD45-PE-Cy antibody (BD Biosciences, United States) were added into the test tube, and blank control was set in the fourth tube. Incubate at room temperature and away from light for 30 min, discard the upper liquid after centrifugation, then add 1.0 mL PBS into the test tube, and use flow cytometry (Beckman coulter, Germany) for analysis.

### 2.5 Rat BMSCs induced by rrPDGF-BB

Our previous study found that the concentration was 25 μg/L rrPDGF-BB (Catalog: 520-BB, R&D Systems, United States) has the best effect in inducing rat BMSCs for 3 days (Jiang et al., 2021a; Wei et al., 2021a; Jiang et al., 2021b; Wei et al., 2021b). The concentration of rrPDGF-BB configured in this study is 25 μg/L conditioned medium, use this conditioned medium to culture the third generation BMSCs, and observe the cells under light microscope (ZEISS Axio observer Z1, ZEISS, Germany).

### 2.6 Preparation of cell injection

Cultivate the BMSCs mentioned above, and when they fill the bottom of the culture bottle, fully digest them with trypsin, and prepare them into cell suspension with a concentration of 1 × 10^6^/mL。 BMSCs cell suspension without induction was prepared by the same method for standby.

### 2.7 Model of distraction osteogenesis

**FIGURE 2:**
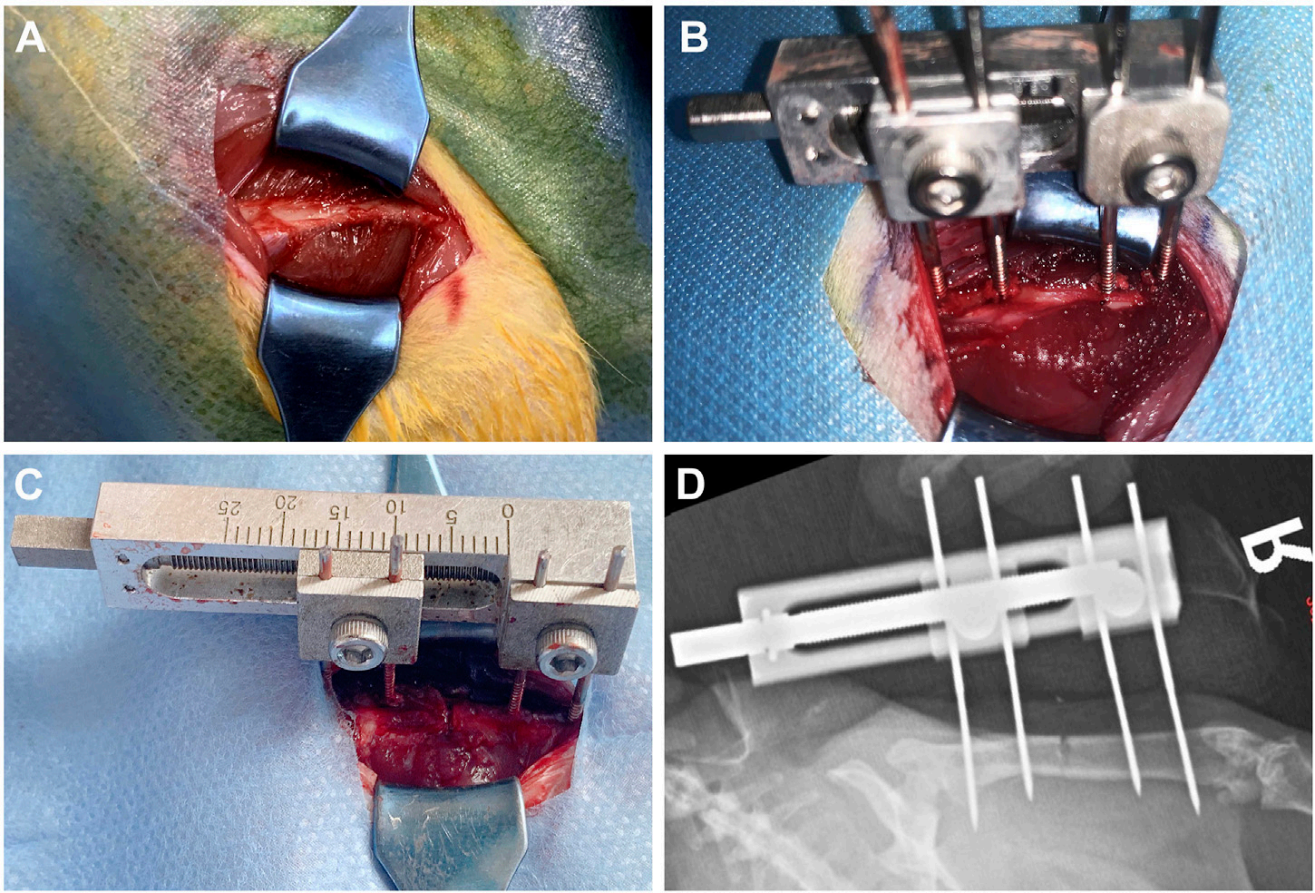
Schematic diagram of the operation of rat right femoral distraction osteogenesis model. **(A-C)** The surgical procedure. **(D)** X-ray examination after operation.

Rats were fasted for 6 h before operation. 350–400 g male SD rats were selected and anesthetized with 2% pentobarbital sodium. After the anesthesia took effect, the rats were placed in the right lateral position. The operation area was shaved with an animal razor. After shaving, the operation area was disinfected with skin iodine for three times and sterile sheets were laid. A 3.0 cm strip incision was taken at the lateral side of the right femur of the rat. After the skin was cut according to the femoral course of the rat, a white line was visible, and the subcutaneous tissue and fascia were cut layer by layer (Figure 2A). Use vascular forceps to separate the surrounding muscles and other soft tissues layer by layer, and use hooks to pull the surrounding tissues to protect the nerves, blood vessels, muscles, etc. A titanium alloy Kirschner wire with a diameter of 1.0 mm was drilled into the proximal femur perpendicular to the femur, marked as No. 1; Use the nail path of the external fixation device to match the corresponding position of the femur, and drill No. 3 Kirschner wire into the distal femur; Using the same method, drill in No. 2 and No. 4 Kirschner pins. After fixing firmly, install the external fixing device and adjust the external fixing device to a proper position (Figure 2B). At this time, pull the surrounding tissues with a draw hook to protect the soft tissues in the operation area. Pay attention to protecting the periosteum. At the middle of No. 3 and No. 4 Kirschner wires, cut the femur with a pendulum saw (YTZJ-B, Zhengda, Hangzhou, China). During osteotomy, wash the osteotomy area with low temperature normal saline to avoid thermal damage during osteotomy (Figure 2C). Use an internal square wrench to turn the nut of the external fixation device clockwise. The two ends of the femur are separated from each other under direct vision, indicating that the femur is completely disconnected, and the molding is successful (Wu et al., 2020; Liu Y. et al., 2021; Wu et al., 2021; Liu et al., 2022). Rinse the wound with normal saline, suture the wound layer by layer, bind it with sterile gauze, and complete the modeling.

### 2.8 Postoperative treatment

The rats were given normal diet 6 h after the operation, and were given painkillers and antibiotics to prevent infection until the third day after the operation. The wound was wiped with iodophor cotton balls at an interval of 2 days after surgery, and clean sterile gauze was replaced until the wound healed. When severe wound infection was found, debridement was performed on the infected rat wound in time.

### 2.9 Distraction process

**FIGURE 3:**
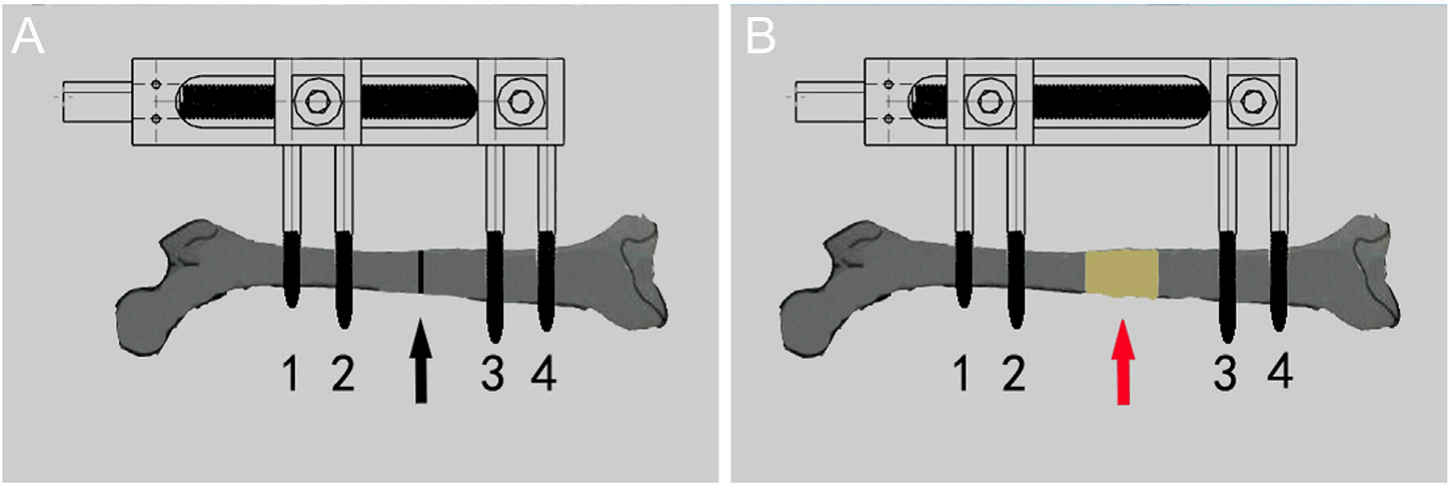
Diagram of the process of distraction osteogenesis in rats. **(A)** Before distraction the femur (black arrow shows osteotomy area). **(B)** After distraction the femur (red arrow shows the distraction area).

After operation, every rat was raised in a single cage. The first 7 days were the rest period. After the rest period, the rats entered into the stretching period. Every 12 h, the internal square wrench was used to rotate the nut of the external fixation device 180° clockwise. The tensioning frequency was 0.25 mm/12 h (Figure 3). The distraction period was 14 days, and the expected distraction was 7.0 mm. After the end of the distraction, the scale of the external fixation device was observed. All rats basically obtained the expected distraction distance, and then the rats entered the mineralization period, Continue single cage feeding until the end of the experiment.

### 2.10 Cell injection into rats

**FIGURE 4:**
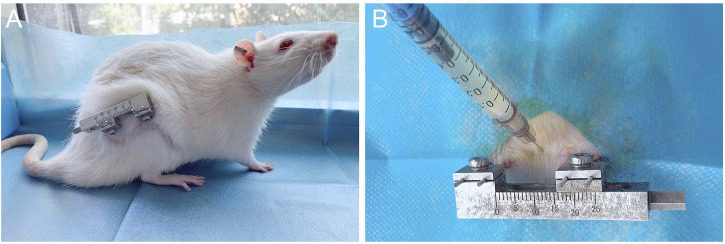
Post-operative picture of the rat and schematic diagram of injected cells. **(A)** Post-operative photos of rats with external fixation devices. **(B)** Schematic diagram of injected of cells into the distraction osteogenesis area of rat femur.

72 rats were randomly divided into three groups; Control group: 0.3 mL PBS injection; group 1:0.3 mL BMSCs cell suspension was injected. Group 2:0.3 mL of BMSCs cell suspension induced by rrPDGF-BB was injected. Inject cells once on the first day of mineralization, the injection method: the rats were anesthetized and the injection area was disinfected. 0.3 mL cell suspension (the concentration was 1 × 106/mL) was drawn with a 1.0 mL syringe, and the needle of the syringe was inserted into the distraction area. There was a sense of penetration. No bleeding was found after the pump back, and then the cells were injected into the distraction area. The syringe was removed and the wound was pressed for 2min without significant bleeding, then disinfected again and wrapped in a sterile dressing (Figure 4B).

### 2.11 X-ray examination

X-ray was taken on all rats on the day after operation, after the end of distraction and at 2, 4, and 8 weeks of mineralization (Kodak DR7500, Department of Radiology, First Affiliated Hospital of Xinjiang Medical University, Urumqi, China).

### 2.12 Micro-CT examination

At the 8th week of mineralization period, three rats in each group were killed at random, and bilateral femoral specimens were taken. After cleaning the specimens, Micro-CT examination (Scanco Medical, Switzerland). Micro-CT program was used to calculate the data of each group, including the tissue volume, bone volume, percent bone volume, bone trabecular thickness, bone trabecular number, bone trabecular separation and bone mineral density, and statistical analysis was conducted.

### 2.13 Sample collection

At 2, 4, and 8 weeks of mineralization period, six rats in each group were randomly selected and sacrificed to collect femur specimens. The sample was fixed with 4% paraformaldehyde (Solarbio, Beijing, China) for 48 h, and then cleaned with PBS. After cleaning, pour in 10% EDTA decalcification solution (Solarbio, Beijing, China). It was estimated that decalcification will last for 4 weeks (Savi et al., 2017; Bogoevski et al., 2019; Dou et al., 2021; Alaeddini et al., 2022). When the samples was elastic, it indicated that decalcification was complete, and the samples was stored in 75% alcohol.

### 2.14 Histomorphological analysis

The decalcified samples were cleaned and then gradually dehydrated with alcohol, after which they were immersed in xylene (Sigma, Switzerland) and finally embedded in paraffin (Leica, Germany) (Guo et al., 2020; Nakamura et al., 2021; Marinopoulos et al., 2022). The samples were frozen at −20°C for 8 h, and then cut into 3.0 μm thick sections with a microtome (RM2135, Leica, Germany), removed with adhesion microscope slides (Citotest, Jiangsu, China) and baked in a 60°C oven.

### 2.15 HE staining

The slides containing the samples were hydrated step by step, dipped in hematoxylin dye solution (Sigma, Switzerland) for staining, washed with ultrapure water and then dipped with 1% hydrochloric acid ethanol (Sigma, Switzerland), washed again with ultrapure water and then re stained with eosin solution (Sigma, Switzerland), and the xylene was transparent after alcohol dehydration. Finally, cover the glass slide, seal the slides with neutral resin (Solarbio, Beijing, China), and observe under the light microscope after the slices were dried.

### 2.16 Statistical method

SPSS 26.0 statistical software was used for statistical analysis of data. The data were expressed by mean ± standard deviation (Mean + SD). Analysis of variance was used for comparison between groups. *p* < 0.05 was statistically significant.

## 3 Result

### 3.1 Morphological observation of rat BMSCs

**FIGURE 5:**
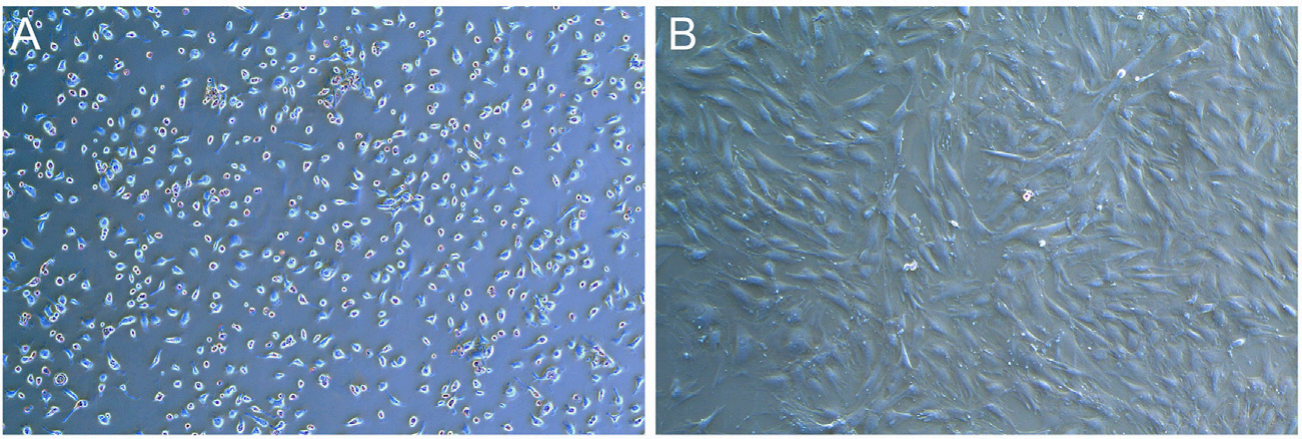
Photos of BMSCs in SD rats (X50). **(A)** Photos of primary cells. **(B)** Photos of the third generation cells.

Primary rat BMSCs adhered to the wall and grew as small, round or oval cells with regular morphology and slow cell growth (Figure 5A). After passage, the cells grew faster and began to spread in a spindle shape and round shape. In the third generation, cells proliferate in a swirl. The cells in the center of the whirlpool were dense, large in size, clear in outline, mostly flat spindle or irregular, and 1-3 nucleoli could be seen in the cells under high magnification (Figure 5B).

### 3.2 Purity identification of rat BMSCs by flow cytometry

**FIGURE 6:**
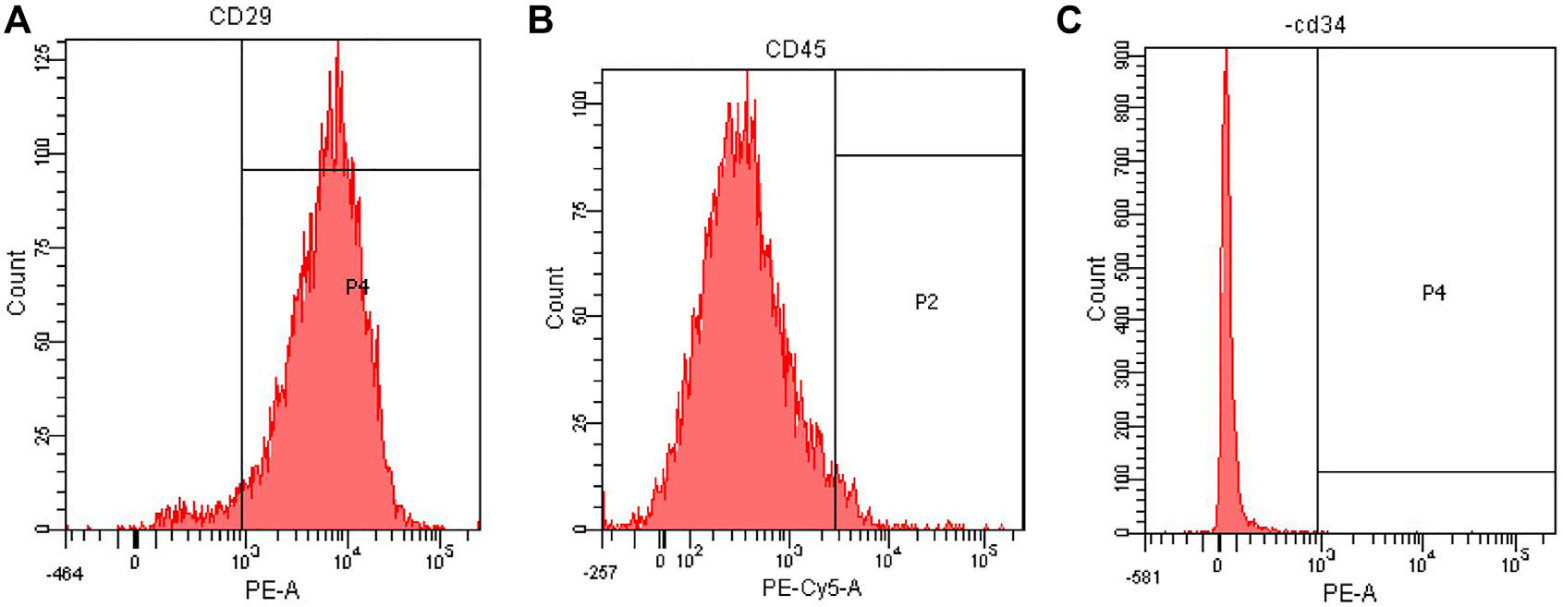
Flow cytometry analysis of rat BMSCs. **(A)** Expression of CD29. **(B)** Expression of CD45. **(C)** Expression of CD34.

Flow cytometry was used to detect the surface markers of BMSCs. The results showed that the third generation BMSCs expressed high CD29 (95.0%), low CD45 (3.6%) and CD34 (0.1%), the purity of BMSCs was high (Figure 6). The results were consistent with those reported in the literature (Cui et al., 2018; Zhao et al., 2018; Yang H. et al., 2020; Liu M. et al., 2021).

### 3.3 Observe induced cells

**FIGURE 7:**
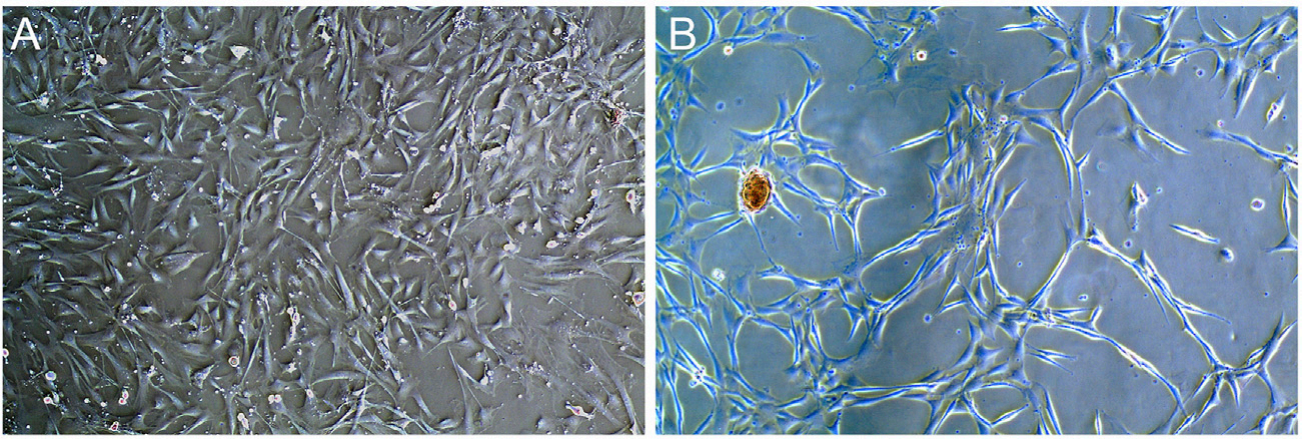
Photos of rrPDGF-BB induced BMSCs (X50). **(A)** One days after intervention. **(B)** Three days after intervention.

After the rat BMSCs of third generation were cultured in the medium containing rrPDGF-BB, the culture medium contained floating cells and cell fragments under the microscope on the first day of induction, which decreased after fluid change. The cell morphology changed from the initial oval shape to an elongated shape with no significant change in cell volume (Figure 7A). On the third day of induction, some cells died. Under the microscope, the cells were reduced in size and elongated in shape, showing a flat spindle shape. The cells were arranged in a circular or reticulated arrangement, with some cells growing together (Figure 7B).

### 3.4 Observation of rat activity

During the experiment, the spirit of the rats was good, the diet and sleep were good, and the activities were good. At the third week after operation, there was a strip scar on the lateral side of the right femur of the rats, which was well healed. The hair around the wound had grown and completely covered the wound (Figure 4A). When the skin around the wound was touched, the rats responded. The skin temperature was acceptable, and there was no leakage at the suture. The external fixation device was well fixed, and four titanium alloy Kirschner wires were vertically drilled into the femur, without looseness and falling off. The skin around the Kirschner wire had no inflammatory reaction such as pus overflowing, and the wound had crusted and healed well. The external fixation device was gently moved, and it was found that the fixation was good without loosening or displacement. The rats were gently placed on the ground, and the rats walked well with a slight sense of load. The knee joint and ankle joint of the rats were active, and the rest were normal.

### 3.5 X-ray examination

**FIGURE 8:**
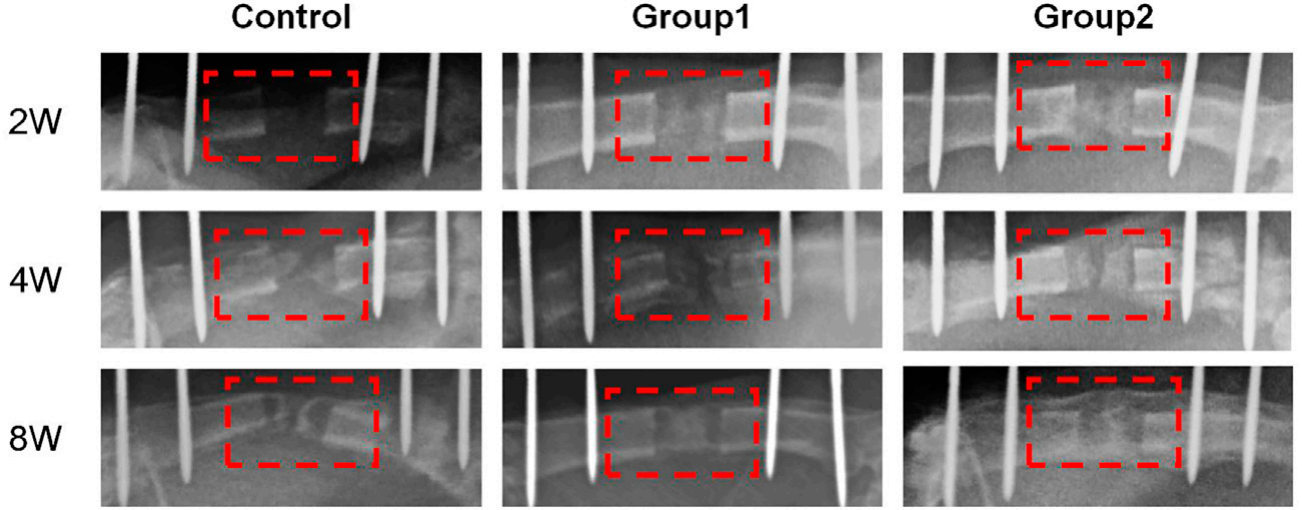
X-ray photos of femoral samples at different time points.

The X-ray examination was taken on the day after the operation, which showed that the femoral shaft was completely separated, and the alignment of the broken end was good (Figure 2D). The X-ray examination taken after the end of the distraction showed that the distraction had reached the expected length. During the distraction, the osteotomy end was gradually separated without dislocation, and there was no obvious callus formation in the distraction area. On the 14th day of mineralization period, callus formation was observed in the distraction area of groups 1 and 2, with uneven new bone formation and no continuous callus, and the density of callus in group 2 was higher than that in group 1. In the control group, the distraction area was bright without obvious callus formation. The osteotomy ends of the three groups were flat and clearly demarcated from the normal bone. On the 28th day of the mineralization period, callus formation was observed in the stretch area of the three groups, and the new callus was strip like or sheet like new bone, and the callus density in group 2 was greater than that in group 1, while the callus density in the control group was the smallest. The new bone in the distraction area of the three groups was cloudy, with low density, relatively continuous bone, uneven osteotomy end, and obvious boundary with normal bone. On the 56th day of mineralization period, uniform and continuous callus could be seen in the distraction area of groups 1 and 2 without obvious bright area, and the callus density of group 2 was higher than that of group 1, while the density of control group was the lowest. In group 2, the stretch area was dense and uniform, the bone was continuous, the osteotomy end was uneven, and the boundary with normal bone was blurred. In group 2, the bone marrow cavity was seen, but no bone marrow cavity recanalization was observed. The osteotomy ends of the control group and group 1 weren’t smooth and had a clear boundary with the normal bone. In the control group, the new bone was discontinuous and there were transparent areas. The three groups of X-ray examination did not find the phenomenon of femoral bone non-union, bone defect and soft tissue incarceration (Figure 8).

### 3.6 Micro-CT examination

**FIGURE 9:**
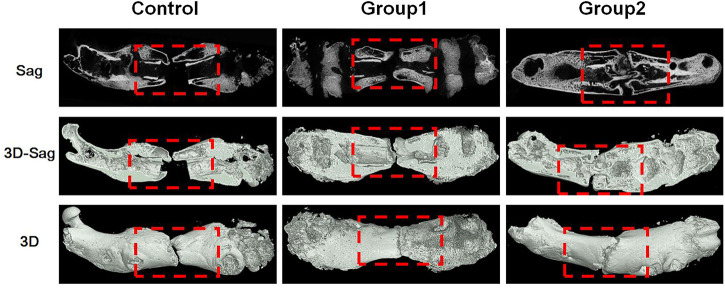
Micro-CT images of femurs in each group at the 8th week.

At the 8th week, the femur specimens of rats in each group were taken, and new bone was formed in the stretch area of the three groups, and the femurs were thickened. In the control group, the healing was poor, a small amount of callus was formed, and the broken end was not connected, and there was a certain gap that was not healed; In group 1, a large amount of callus was formed in the distraction area, and the broken end was partially connected, and no bone marrow cavity was reopened; In group 2, the healing was good, there were more callus in the distraction area, and the disconnected ends were connected, but no bone marrow cavity was reopened (Figure 9).

### 3.7 Micro-CT parameter analysis

**FIGURE 10:**
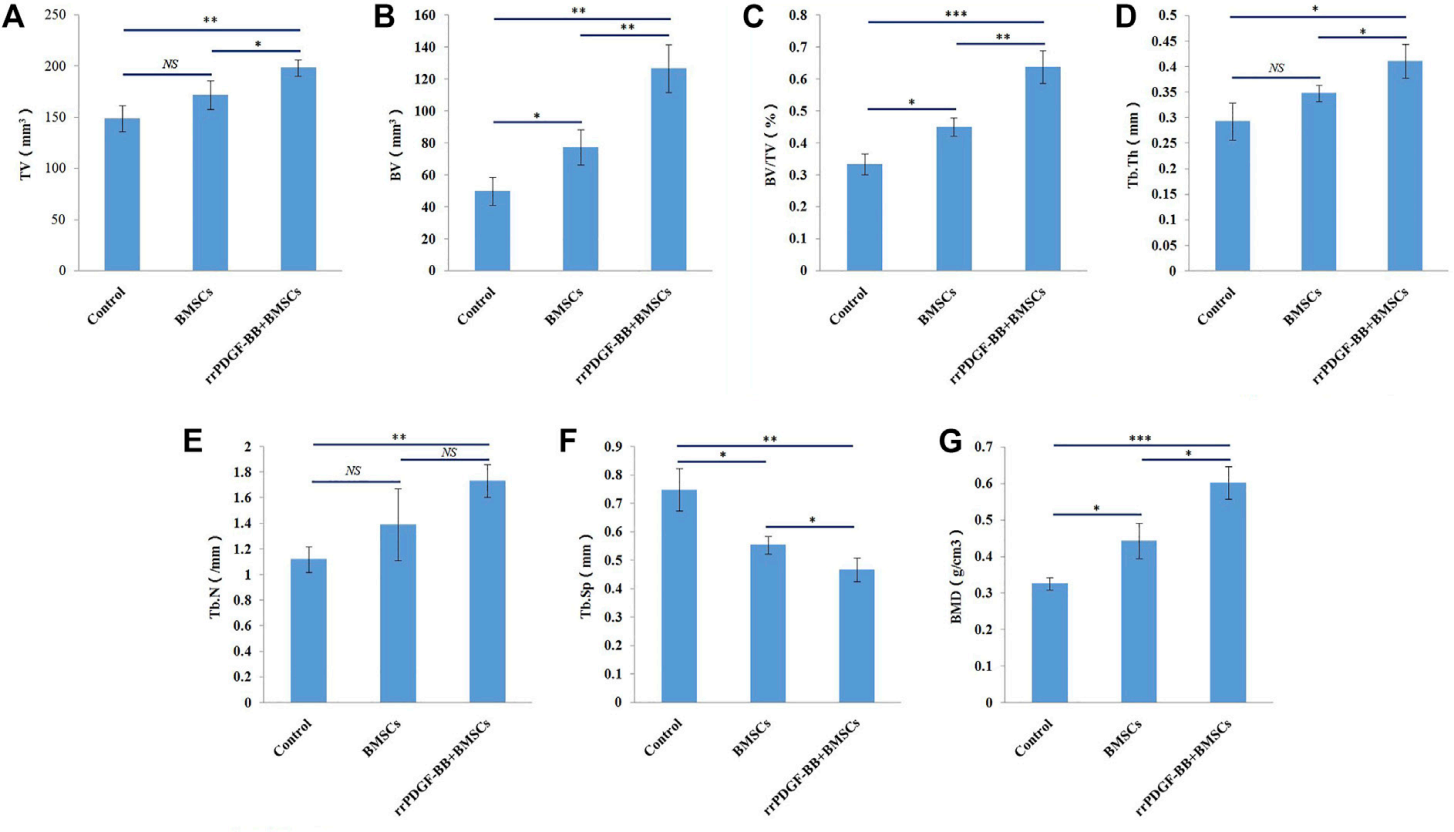
Micro-CT parameters of rat femora at week 8. **(A)** Micro-CT analyses of tissue volume (TV) in each group. **(B)** Micro-CT analyses of bone volume (BV) in each group. **(C)** Micro-CT analyses of percent bone volume (BV/TV) in each group. **(D)** Micro-CT analyses of trabecular thickness (Tb.Th) in each group. **(E)** Micro-CT analyses of trabecular number (Tb.N) in each group. **(F)** Micro-CT analyses of trabecular separation (Tb.Sp) in each group. **(G)** Micro-CT analyses of bone mineral density (BMD) in each group. ***p-value <0.001. **p-value <0.01. *p-value <0.05.

The Micro-CT parameters of rat femur specimens were shown in Table 1 and Figure 10. The tissue volume, bone volume, percent bone volume, bone trabecular thickness, bone trabecular number and bone mineral density of group 2 were the largest, followed by group 1, and the control group were the smallest, with statistical difference between groups (*p* < 0.05). The bone trabecular separation in the control group was the highest, followed by group 1 and group 2, and there were statistical differences between groups (*p* < 0.05).

**TABLE 1:** Micro-CT parameters of rat femora at week 8 (Mean + SD).

Sample	TV (mm^3^)	BV (mm^3^)	BV/TV (%)	Tb.Th (mm)	Tb.N (1/mm)	Tb.Sp (mm)	BMD (g/cm^3^)
Control	148.668 ± 12.893	49.916 ± 8.701	0.334 ± 0.032	0.293 ± 0.036	1.120 ± 0.099	0.749 ± 0.075	0.326 ± 0.017
Group1	171.605 ± 13.900	77.498 ± 10.944	0.450 ± 0.029	0.348 ± 0.016	1.392 ± 0.279	0.554 ± 0.031	0.443 ± 0.049
Group2	198.315 ± 7.881	126.712 ± 14.820	0.638 ± 0.051	0.411 ± 0.033	1.734 ± 0.128	0.467 ± 0.042	0.602 ± 0.045
*F*	13.181	32.812	46.866	11.936	8.164	22.614	36.375
*p*	0.006	0.001	0.000	0.008	0.019	0.002	0.000

### 3.8 General observation of samples

**FIGURE 11:**
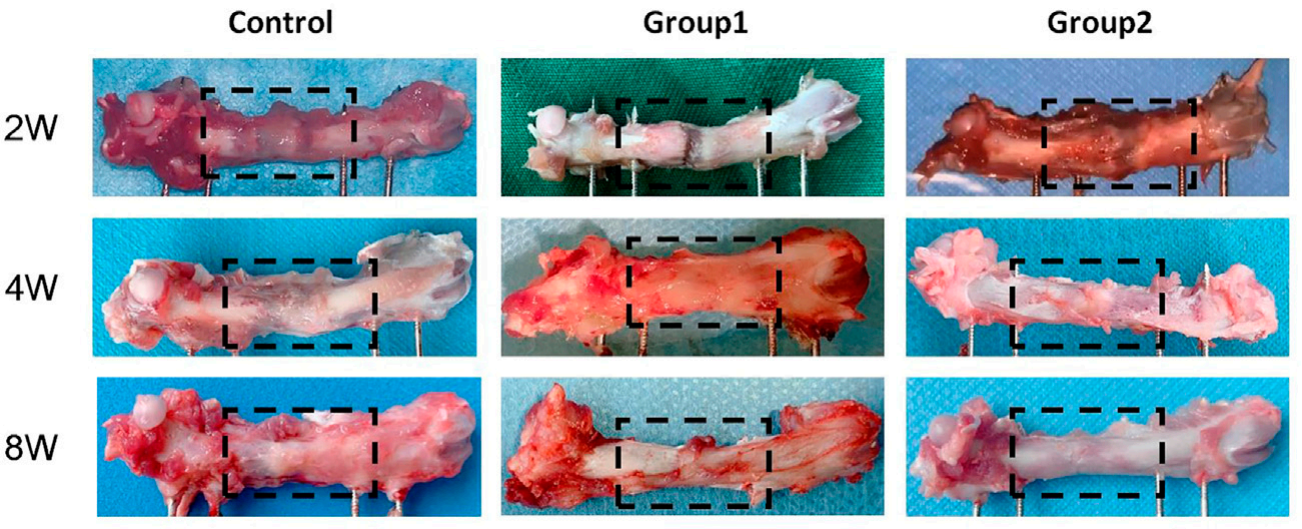
General picture of rat femur.

Femur samples at different time points were placed in a sterile sheet for observation (Figure 11). With the increase of time, the color of the distraction area gradually changed from brown to gray white, and the texture gradually became hard. On the 14th day of the mineralization period, the distraction area of the three groups had a clear boundary with the normal bone tissue, and the femur was elastic. With a little pressure, the femurs could be bent. The distraction area of the control group was brown red with soft texture, while the distraction area of groups 1 and groups 2 was brown with harder texture than the control group, and the distraction area of group 2 was harder than that of group 1. On the 28th day of the mineralization period, the new bone tissue in the distraction area of the control group was brown, with a clear boundary with the normal bone tissue, and the distraction area of group 1 and group 2 was gray white; The hardness of the new bone tissue in the distraction area of the three groups increased compared with that before. The hardness of group 2 was the highest, followed by group 1, and the hardness of the control group was the lowest. At the 56th day of mineralization, the distraction area of the control group was grayish white, with a clear boundary with normal bone tissue; In group 1 and group 2, the distraction area was white, with no obvious boundary with normal bone tissue; The hardness of the new bone tissue in the distraction area of the three groups was higher than before, the hardness of group 2 was the highest, followed by group 1, and the hardness of the control group was the lowest; In group 2, the texture of the distraction area was hard and the elasticity was poor. When the femur was bent, the femur was not easy to bend.

### 3.9 HE staining

**FIGURE 12:**
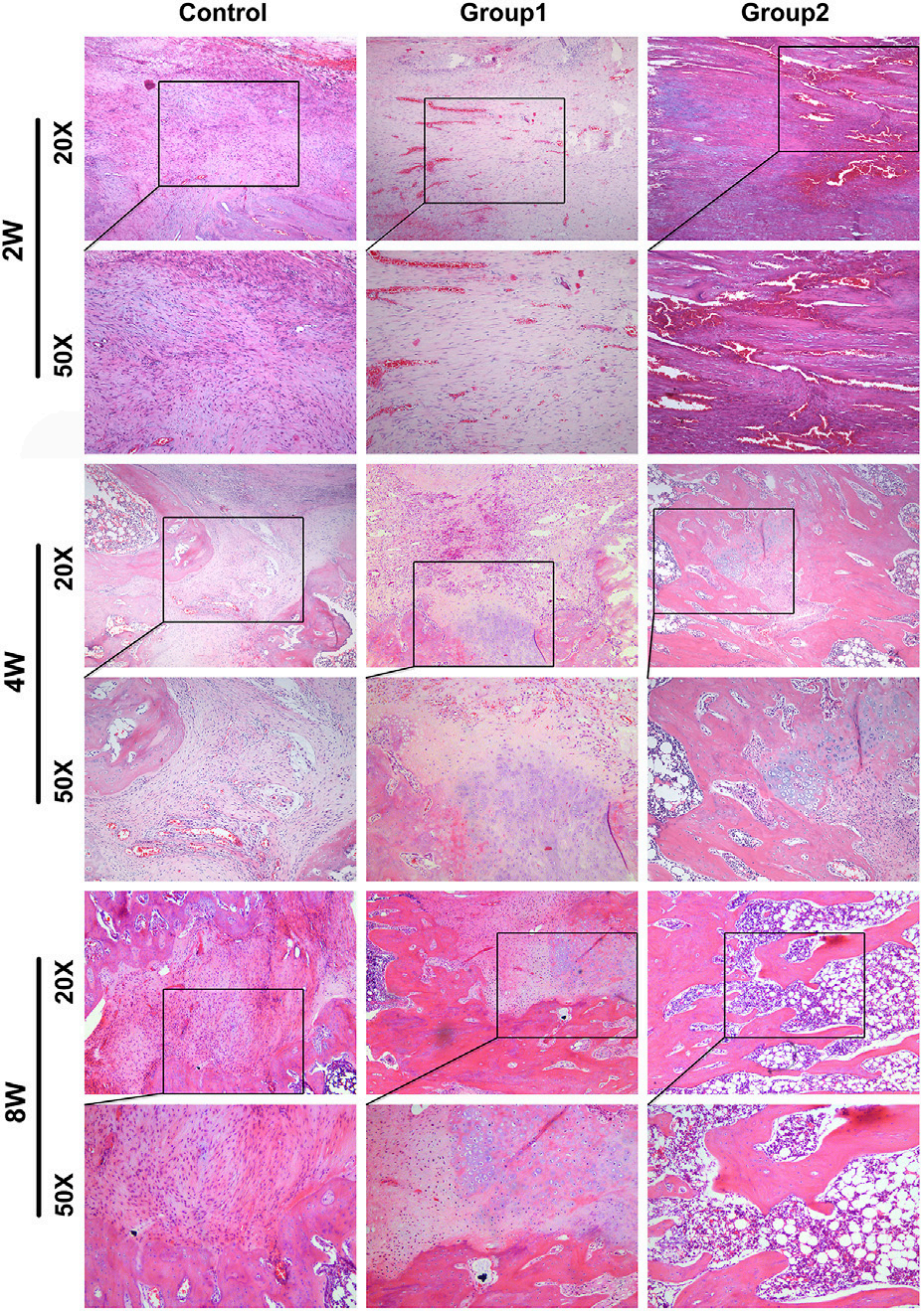
HE staining photos of femoral samples at different time points (X50).

Histological observation was carried out on rat samples of different groups at 2, 4, and 8 weeks respectively (Figure 12). At 2 weeks, fibroblasts and new blood vessels were mainly found in the distraction area. The most new blood vessels were found in the distraction area of group 2, followed by group 1. In the control group, fibroblasts were mainly seen under microscope, and new blood vessels were few; In addition, a small amount of chondrocytes were generated in the distraction area of group 2. In the 4 weeks control group, fibroblasts were dominant under microscope, and the number of new blood vessels increased compared with that before; In group 1, fibroblasts were mainly seen under microscope, but the number was less than before, and there were new chondrocytes; In group 2, the new bone cells were mainly in the distraction area, the number of fibroblasts was less, and the number of chondrocytes was more than before. In the 8-week control group, the majority of the specimens were fibroblasts under microscope, and the number of new bone tissue was higher than before; group 1 was mainly composed of new bone tissue under microscope, with more chondrocytes and fewer fibroblasts than before. The new bone tissue had been connected into pieces and formed a few bone marrow cavities; In group 2, new bone tissue was mainly seen under microscope, no chondrocytes and fibroblasts were found, and bone marrow cavity was seen under microscope, part of which had been fused.

## 4 Discussion

Bone healing is a highly coordinated process, which is jointly affected by various growth factors and cells. Tissue engineering uses exogenous or endogenous growth factors, seed cells and scaffolds to simulate these healing processes. Many studies have described the effects of single factor and multi factor applications on bone healing (Hamushan et al., 2020; Shen et al., 2021; Ye et al., 2021; Han et al., 2022). Our study showed that injecting rrPDGF-BB into the distraction space during the mineralization period of distraction osteogenesis could promote the formation of new bone in the distraction area and promote healing.

In order to promote the healing of distraction osteogenesis, researchers in various countries have conducted a large number of experimental studies on it. Akçay found that the systemic application of vitamin E can stimulate the formation of new bone in rabbit distraction osteogenesis, thereby shortening the treatment time (Akçay et al., 2019); Acikan found that systemic application of melatonin can increase the formation of new bone in distraction osteogenesis (Acikan et al., 2018); Altay found that oxytocin can promote bone metabolism, increase new bone formation and promote bone healing in the distraction area (Altay et al., 2020); McDonald applied sclerostin antibody (Scl-Ab) in the rat model of distraction osteogenesis. The results showed that Scl-Ab treatment could promote bone formation and lead to bone regeneration with larger volume and higher strength. They believed that Scl-Ab had potential clinical value in preventing re fracture (McDonald et al., 2018); Jia found that mesenchymal stem cell exosomes can enhance the proliferation and osteogenesis of BMSCs, and promote the bone regeneration of distraction osteogenesis in aged rats (Jia et al., 2020). Tissue engineering is a better method to promote regeneration, so the combination of bone tissue engineering and distraction osteogenesis can promote the development of distraction osteogenesis (Yang et al., 2017; Roddy et al., 2018; Valtanen et al., 2021).

Some scholars had confirmed that BMSCs could be recruited into the distraction space, and then differentiated into osteoblasts or chondrocytes (Ai-Aql et al., 2008; Dhaliwal et al., 2016). This process was accompanied by callus formation (Wang et al., 2013; Yuan-Zhe and Lee, 2018), so BMSCs transplantation was a valuable regenerative therapy. Previous studies have shown that vasculature is the source of PDGFB. Vasculature plays a critical role in osteogenesis and bone diseases (Chen et al., 2021; Owen-Woods and Kusumbe, 2022), and vascular PDGF signalling is involved (Singh et al., 2019). Studies had confirmed that PDGF-BB could promote cell mitosis, chemotaxis of fibroblasts and BMSCs, promoted angiogenesis, promoted bone healing, etc., (Gruber et al., 2003; Ozaki et al., 2007). Our previous study found that the distraction space was gelatinlike tissue, and under the microscope, it was newly generated fibroblasts, new blood vessels, etc., (Wu et al., 2020). The new tissue in the distraction space can act as a scaffold material. Therefore, we believe that exogenous supplement of BMSCs and PDGF-BB can achieve the purpose of promoting healing.

After BMSCs were transplanted into the body, the differentiation was poor, the survival rate was low, and the regeneration potential was limited (Ortinau et al., 2019); Bowenpope made a rat model of distraction osteogenesis, and injected rhPDGF-BB of different density into the distraction area to verify the therapeutic effect of rhPDGF-BB on rat distraction osteogenesis (Bowenpope et al., 1984). However, the half-life of PDGF-BB is very short, and only continuous injection can achieve the purpose of treatment, but it’s expensive (Moore et al., 2009); In addition, there were problems such as fluid leakage during injection, so our research group first used rrPDGF-BB to intervene BMSCs, and then transplanted them. In addition, our previous *in vitro* experiments had confirmed that rrPDGF-BB of 25 μg/L significantly promoted differentiation of BMSCs, and once the concentration exceeded 50 μg/L, it would lead to cell death.

In our experiment, after we used rrPDGF-BB to induce with BMSCs, the cell morphology changed, and a circular or reticular structure appeared. We speculated that the cells had differentiated and had a preliminary vascular rudiment, which was consistent with the report of Heldin (Heldin and Westermark, 1999; Ozaki et al., 2007), and our previous research confirmed that PDGF-BB could promote the proliferation of rat BMSCs and induce BMSCs to differentiate into vascular endothelial cells (Jiang et al., 2021a; Jiang et al., 2021b), which was basically consistent with the results of this study. Then we transplanted the BMSCs induced by rrPDGF-BB into the distraction osteogenic area of rats, and then used general observation of samples, X-ray examination, Micro-CT examination and histological staining to verify its therapeutic effect. The results confirmed that injecting the BMSCs induced by rrPDGF-BB into the rat distraction space can promote the formation of new bone in the distraction area, which is consistent with the report of Moore (Moore et al., 2009; Del Rosario et al., 2015; Luvizuto et al., 2016; Gao et al., 2021; Komatsu et al., 2022). Moore studied the effect of PDGF-BB on bone repair, so Moore directly injected PDGF-BB into rats. Different from Moore, We studied the effect of BMSCs induced by PDGF-BB on bone repair, and our previous research confirmed that PDGF-BB can induce BMSCs to differentiate into osteoblasts (Wei et al., 2021b). In addition, some scholars showed that the effect of injecting stem cells in the distraction period had a better curative effect than the injection of stem cells in the mineralization period (Yang Y. et al., 2020). Therefore, choosing the appropriate dose of PDGF-BB to intervene in BMSCs and injecting BMSCs in the appropriate period will bring greater benefits, which is also our future research direction.

In conclusion, our study shows that injecting the BMSCs induced by rrPDGF-BB into the rat distraction space during the mineralization period of distraction osteogenesis can promote bone regeneration and healing. The results of this study have certain clinical value. However, whether BMSCs induced by PDGF-BB have differentiated *in vivo* has not been explored in this topic. Later, we will continue the research in this area, screen out the appropriate induction dose and time, and reduce the incidence of complications, so as to make further contributions to the research of distraction osteogenesis.

## Data Availability

The original contributions presented in the study are included in the article/supplementary material, further inquiries can be directed to the corresponding author.
